# Occurrence of Hemotropic Mycoplasmas in Commercial Pig Herds in Southeastern Brazil

**DOI:** 10.3390/microorganisms13061328

**Published:** 2025-06-07

**Authors:** Daniele Soares Fialho, Agostinho Sérgio Scofano, Karyne dos Santos Marins da Silva, Katielle Ribeiro da Silva, Lara Celeste Araujo do Carmo Cordeiro, Nathalie Costa da Cunha, Elmiro Rosendo do Nascimento, Thomas Salles Dias

**Affiliations:** 1 Programa de Pós-Graduação em Medicina Veterinária (Higiene Veterinária e Processamento Tecnológico de Produtos de Origem Animal), Universidade Federal Fluminense, Rio de Janeiro 24220, Brazil; daniele_soares@id.uff.br (D.S.F.); karynemarins@id.uff.br (K.d.S.M.d.S.); elmirorosendo@id.uff.br (E.R.d.N.); 2 Instituto de Defesa Agropecuária e Florestal do Espírito Santo, Vitória 29000, Brazil; agoscofano@gmail.com; 3 Departamento de Saúde Coletiva Veterinária e Saúde Pública, Universidade Federal Fluminense, Rio de Janeiro 24220, Brazil; kribeiro@id.uff.br (K.R.d.S.); lceleste@id.uff.br (L.C.A.d.C.C.); nathaliecunha@id.uff.br (N.C.d.C.)

**Keywords:** *Mycoplasma suis*, hemotropic mycoplasmas, swine production, molecular diagnosis

## Abstract

*Mycoplasma suis* infects pig red blood cells and is linked to anemia, weakened immunity, and production losses. Infected animals may remain subclinical carriers, contributing to pathogen dissemination. This study aimed to investigate the prevalence of *M. suis* in commercial pig farms in the state of Espírito Santo, Brazil. A total of 416 blood samples from 55 farms were analyzed using conventional PCR targeting the hemotropic Mycoplasmas (16S rRNA) and a species-specific PCR for *M. suis* (23S rRNA). Among the samples, 131 (31.49%) tested positive for hemoplasmas and 58 (13.94%) for *M. suis* with a significantly higher frequency in sows (*p* < 0.01). The Metropolitan microregion showed the highest prevalence (23.53%). The discrepancy between the prevalence for the genus and the species suggests the possible presence of other hemotropic *Mycoplasma* species and highlights the limitations of 16S rRNA-based assays. These findings emphasize the need for more specific molecular targets and continuous monitoring strategies to control infection in pig farming.

## 1. Introduction

Swine production plays a crucial role in the Brazilian economy, with Brazil ranking as the fourth largest producer and exporter of pork worldwide [1]. The sector has undergone significant modernization, adopting advanced technological practices to improve productivity and ensure herd health. Nevertheless, the presence of infectious diseases remains a major challenge to sustainable production. Among the pathogens that negatively impact the sector, hemotropic Mycoplasmas, particularly *Mycoplasma suis*, represent a significant threat to animal health and production efficiency [2,3,4,5].

Hemoplasmas have been reported affecting both production and companion animals across different regions of the world [6]. Subclinical infections are frequently observed and may result in reduced weight gain, decreased feed efficiency, and increased susceptibility to secondary infections [7,8]. Hemoplasmas can act as opportunistic agents, exacerbating clinical conditions in immunosuppressed animals or those under environmental stress [9]. *Mycoplasma suis* is a pleomorphic, cell wall-deficient microorganism that parasitizes porcine erythrocytes, potentially causing hemolytic anemia, jaundice, fever, and lethargy. In acute cases, these signs may progress to severe hemolytic anemia, while chronic infections are characterized by moderate anemia, vasculitis due to vascular damage, hemorrhagic diathesis, infertility, and immunosuppression [2,7,10]. Infection with *M. suis* is associated with chronic anemia, immune system impairment, and negative impacts on swine reproduction and growth [2]. Furthermore, pigs infected with *M. suis* may remain chronic carriers even after the resolution of clinical signs [5]. These consequences directly compromise zootechnical performance, resulting in significant economic losses for producers.

Molecular diagnosis has been widely employed for the identification of hemoplasmas in swine, with the 16S rRNA gene being commonly targeted in PCR assays due to its conservation across different bacterial species [9,11]. Despite its effectiveness for genus-level screening, the high similarity between species such as *M. suis* and *M. parvum* may compromise test specificity [12,13]. To overcome this limitation, the 23S rRNA gene has been utilized as an alternative target, offering greater discriminatory power between closely related species, particularly for the specific detection of *M. suis* [13,14].

Investigating the prevalence of *M. suis* in commercial pig farms is essential to understand the agent’s distribution and the associated risk factors. In this context, studying the prevalence and impacts of *M. suis* infection is of great importance for Brazilian swine production. Continuous herd health monitoring, combined with the use of molecular diagnostic tools, may assist in the development of more effective sanitary policies, reducing economic losses and promoting sustainable production. Understanding the infection dynamics and the factors influencing its spread is crucial to mitigate its effects and to ensure animal productivity and welfare. Thus, the aim of this study was to investigate the prevalence of *M. suis* in commercial swine farms from the state of Espírito Santo, Brazil.

## 2. Materials and Methods

### 2.1. Farm Selection and Sampling Design

A total of 55 commercial swine farms located in the state of Espírito Santo, Brazil, were included in this study. Participation was voluntary, with prior informed consent obtained from all producers. All participants signed a written informed consent form after receiving detailed information regarding the research objectives, data confidentiality, and their right to withdraw from participation at any time. Farm selection was based on a previous survey using records available from the Agricultural Integration System of the Institute of Agricultural and Forestry Defense of Espírito Santo (IDAF-ES), covering approximately 72.4% of the officially registered commercial swine farms in the state. The territorial division of the state into microregions, based on municipal specificities, was adopted to facilitate more efficient planning of future sanitary actions.

To determine the required sample size, an estimated total population of 241,550 pigs was considered [15]. Statistical calculations were performed using Epi Info™ version 7.2.5 software (Centers for Disease Control and Prevention, Atlanta, GA, USA), applying a 50% expected prevalence, a 95% confidence level, and a 5% margin of error. Based on these criteria, the minimum required number of blood samples was estimated at 384. In total, 416 blood samples were collected, ranging from 3 to 10 animals per farm according to herd size, through proportional stratified sampling. Inclusion criteria encompassed sows in culling phase—either mid-gestation or at the end of the farrowing period—and finishing pigs ready for slaughter. Animals with low body weight or undergoing medical treatment were excluded from the sampling to avoid potential biases in the health and microbiological status of the population.

### 2.2. Sample Collection and Processing

The selection of pigs for blood sampling was performed randomly within the herds. Samples were collected in EDTA-containing tubes and kept frozen until laboratory processing. Genomic DNA extraction was carried out using the Wizard^®^ Genomic DNA Purification Kit (Promega), following the manufacturer’s instructions. The extracted DNA was stored at –20 °C until further use in PCR analyses.

### 2.3. Polymerase Chain Reaction (PCR)

The detection of the hemotropic Mycoplasma was performed by conventional PCR, using the primers HemMycop16S-322s (5′-GCCCATATTCCTACGGGAAGCAGCAGT-3′) and HemMyco16S-938as (5′-CTCCACCACTTGTTCAGGTCCCCGTC-3′), as described by Varanat et al. [16]. The thermocycling conditions were as follows: initial denaturation at 94 °C for 4 min, followed by 30 cycles of 94 °C for 30 s, 68 °C for 45 s, and 72 °C for 45 s, with a final extension at 72 °C for 5 min and subsequent storage at 4 °C. The expected amplicon size was 600 bp.

Samples tested positive for the hemotropic Mycoplasma were subjected to a second PCR assay for the species-specific identification of *M. suis*, using the primers MS_23SF1 (5′-GAAGTTTGAGCGAGAGCACAG-3′) and MS_23SR3 (5′-AGGGCTTAAGTTAGAAGCTTCAGC-3′), according to Thongmeesee et al. [13]. The thermocycling conditions were as follows: 94 °C for 4 min, followed by 30 cycles of 94 °C for 30 s, 64 °C for 45 s, and 72 °C for 45 s, with a final extension at 72 °C for 5 min and subsequent storage at 4 °C. The expected product size was 909 bp.

Amplified products were analyzed by electrophoresis on 1.5% agarose gels, stained with ethidium bromide, and visualized under ultraviolet (UV) light. Fragment identification was performed by comparison with a 100 bp molecular weight marker (Ludwig, Brazil).

### 2.4. Statistical Analyses

The prevalence of hemotropic Mycoplasma and *M. suis* was calculated based on the total number of positive samples. The association between animal category (sow or finisher pig) and positivity for *M. suis* was evaluated using Pearson’s Chi-square test (χ^2^), with a significance level of 5% (*p* < 0.05). Observed and expected frequencies were analyzed to determine the contribution of each group to the association. The geographical distribution of positive cases was described by microregion, with infection rates (%) calculated for each area of the state of Espírito Santo.

## 3. Results

Of the 416 blood samples analyzed, 131 were positive for hemoplasmas, resulting in a prevalence of 31.49%. Among these samples positive for the genus, 58 tested positive for the species *M. suis*, corresponding to a prevalence of 13.94% of the total samples and 44.27% of the samples positive for the genus (Table 1). Of the 55 farms evaluated, 21 (38.18%) had at least one positive sample for *M. suis*, indicating the presence of the agent in a significant proportion of the production units.

**Figure 1:**
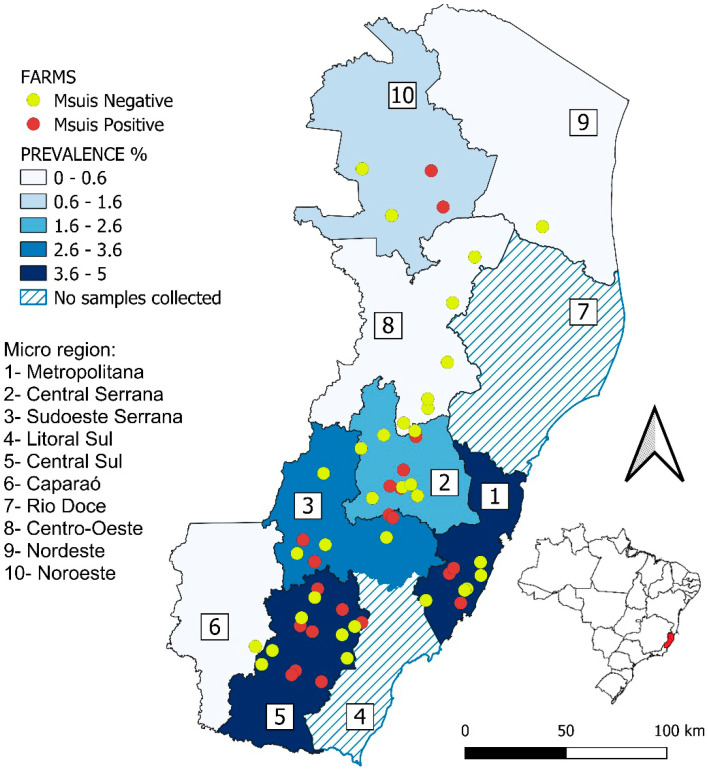
Geographic distribution of the 55 rural properties in the state of Espírito Santo, Brazil, sampled in the study and their *Mycoplasma suis* positivity status.

The geographic distribution of the 55 rural properties sampled in Espírito Santo and their respective *M. suis* status are presented in Figure 1. The Metropolitan microregion showed the highest prevalence for *M. suis* (23.53%). The Southeast Serrana (15.94%) and Central South (14.48%) microregions also presented high prevalences. The Northwest (11.11%) and Central Serrana (10.14%) microregions showed moderate prevalence rates. In contrast, the Caparaó, Central-West, and Northeast microregions did not record any positive cases (Table 2).

The association between animal category (sow or piglet) and *Mycoplasma suis* positivity was statistically significant (χ^2^ = 10.55; *p* = 0.00116; df = 1), indicating that infection rates differed between the groups (Table 3). The prevalence of *M. suis* was significantly higher in sows (16.87%; 56/332) compared to piglets (2.38%; 2/84).

## 4. Discussion

The prevalence of hemoplasmas observed in this study was relatively high, at the animal level, indicating widespread circulation of these agents in the herds analyzed. Cruz et al. [17] reported a similar prevalence (31.9%) in highly technical farms in the Southeastern region of Brazil, which supports the significant presence of these microorganisms even in intensive production systems. Additionally, Gatto et al. [12] described even higher rates, with hemoplasmas detected in over 79% of samples from commercial properties in the Southern region of the country, reinforcing the consistency of these findings across different production contexts.

Although this study did not assess clinical or production outcomes, nor investigate other potential pathogens, the prevalence of *M. suis* detected was notably lower than expected and contrasted with findings from previous studies. For instance, in China, Zhongyang et al. [18] observed an average seroprevalence of 33.3% for *M. suis* with higher incidence in adult pigs, such as sows and breeders. Ngo et al. [19] reported even higher prevalence (86.6%) in farms with a history of reproductive failures in Vietnam. Similarly, Brissonnier et al. [20] detected *M. suis* DNA in 53% of the samples analyzed in South Korea, with none of the farms evaluated being free of infection. These data confirm that *M. suis* is frequently identified as the primary agent involved in hemotropic infections in swine across different production contexts and geographical regions.

The low prevalence of *M. suis* observed in this study, despite the considerable detection of the Mycoplasma genus through screening PCR, raises the possibility of the presence of other hemotropic *Mycoplasma* species. Among them, *Mycoplasma parvum* stands out, a less studied species but already identified in swine herds and associated with subclinical infections, with a tropism for the erythrocyte plasma membrane [17]. Recent studies, such as Ade et al. [21], demonstrated the circulation of M. parvum in clinically healthy pigs in Germany, with a prevalence of up to 36% in finishing pigs and 25% in sows.

This difficulty in differentiating *M. suis* from other hemotrophic *Mycoplasma* species may be partly attributed to the limitations of molecular methods based exclusively on the 16S rRNA gene, which was used in the initial screening. Although commonly employed due to its high sensitivity and ability to detect *Mycoplasma* at the genus level, the 16S rRNA gene shows high sequence similarity among closely related species, such as *M. suis* and *M. parvum*, reducing diagnostic specificity [13]. To address this limitation, more specific molecular targets such as the 23S rRNA gene have been recommended, as they allow for better discrimination between genetically similar species [13,22]. In this study, the use of the 23S rRNA gene increased diagnostic specificity for *M. suis*, but it may have reduced sensitivity compared to broader genus-level detection methods. Such methodological differences, along with environmental and sanitary factors, likely contribute to the variability in *M. suis* prevalence reported across studies.

The observed frequencies were higher than expected in sows and lower in piglets. This pattern suggests greater susceptibility or exposure of sows to the infection, which aligns with studies reporting a higher prevalence of *M. suis* in adult pigs [18,23,24]. This higher prevalence in sows may be associated with multiple factors. Adult animals remain in the production system for longer, increasing their cumulative exposure to the pathogen and vectors. Additionally, physiological stress, particularly during the peripartum period, is a known factor that favors the reactivation of chronic infections, as demonstrated by Hoelzle et al. [2] and supported by more recent studies [9,25]. Evidence suggests that *M. suis* may persist in the body even after treatment and be reactivated during periods of immunosuppression, as observed by Stadler et al. [8]. This behavior reinforces the role of sows as important reservoirs, with the potential to spread the agent within the herd, especially during critical phases of production.

The low prevalence of *M. suis* in piglets may be associated with lower environmental exposure, the presence of maternal antibodies acquired via colostrum, or a short window of infection detection during sampling, as observed by Stadler et al. [24]. Studies suggest that infections in piglets may remain subclinical or latent, only becoming clinically apparent after immunological challenges or stress, such as weaning and changes in the environment [2,20,24].

Some limitations that should be considered when interpreting the results. Firstly, no clinical or productive data were collected from the animals, which limits the ability to establish correlations between *M. suis* infection and clinical outcomes or production losses. Secondly, the molecular investigation was restricted to *M. suis* after the initial genus-level screening; therefore, other hemoplasma species, such as *M. parvum*, may have been present in the population but remained undetected due to the specificity of the second PCR step. Additionally, although the 23S rRNA gene was used to increase diagnostic specificity, it may have reduced overall sensitivity compared to broader detection strategies. Finally, the cross-sectional nature of the sampling provides only a snapshot of the infection dynamics, without capturing seasonal or temporal variations. These limitations highlight the need for future studies with longitudinal designs, the inclusion of clinical data, and broader molecular panels to better understand the epidemiological and pathogenic role of hemotropic Mycoplasma species in swine herds.

Considering these findings and limitations, there is a reinforcement of the need for sanitary surveillance, particularly targeting sows, which present a higher risk of infection and perpetuation of the agent in the herd. Control strategies based on serological and molecular monitoring, associated with improved management practices and stress reduction, are essential for the effective prevention and control of *M. suis* in swine production.

## 5. Conclusions

The low prevalence of *M. suis* observed in this study, in contrast to the high detection of the Mycoplasma genus, may reflect methodological limitations, especially related to the use of less specific molecular targets, such as the 16S rRNA gene. The use of the 23S rRNA gene proved effective for species identification, although with possibly reduced sensitivity. The results reinforce the importance of sows as the primary carriers of the infection and highlight the need for continuous sanitary surveillance strategies, combined with the use of more precise diagnostic tools, to ensure the effective control of the infection and sustainability in swine production.

## Figures and Tables

**Table 1:** Detection of hemotropic Mycoplasma and *Mycoplasma suis* in swine from Espírito Santo.

Results	Hemotropic Mycoplasma	*Mycoplasma suis*
Positives	131	58
Negatives	285	358
Total	416	416
Prevalence (%)	31.49	13.94

**Table 2:** Distribution of *Mycoplasma suis* infection by microregion in Espírito Santo.

Microregion	Sample Size	Number of Positives	Prevalence (%)	
			Microregion	General
Caparaó	10	0	0.00	0.00
Central Serrana	69	7	10.14	1.68
Central Sul	145	21	14.48	5.05
Centro-Oeste	26	0	0.00	0.00
Metropolitana	68	16	23.53	3.85
Nordeste	2	0	0.00	0.00
Noroeste	27	3	11.11	0.72
Sudeste Serrana	69	11	15.94	2.64
Total	416	58	13.94	13.84

**Table 3:** Association between animal category and *Mycoplasma suis* positivity.

Purpose	Negative	Positive	Total
Piglet	82 (97.62%)	2 (2.38%)	84
Sow	276 (83.13%)	56 (16.87%)	332
Total	358 (86.06%)	58 (13.94%)	416

## Data Availability

The data has been contained in the paper.

## References

[1] Associação Brasileira de Proteína Animal–ABPA (2024). Relatório Annual.

[2] Hoelzle L.E. (2008). Haemotrophic Mycoplasmas: Recent Advances in *Mycoplasma suis*. Vet. Microbiol..

[3] Yuan C.L., Liang A.B., Yao C.B., Yang Z.B., Zhu J.G., Cui L., Yu F., Zhu N.Y., Yang X.W., Hua X.G. (2009). Prevalence of *Mycoplasma suis* (Eperythrozoon suis) Infection in Swine and Swine-Farm Workers in Shanghai, China. Am. J. Vet. Res..

[4] Groebel K., Hoelzle K., Wittenbrink M.M., Ziegler U., Hoelzle L.E. (2009). *Mycoplasma suis* Invades Porcine Erythrocytes. Infect. Immun..

[5] Ritzmann M., Grimm J., Heinritzi K., Hoelzle K., Hoelzle L.E. (2009). Prevalence of *Mycoplasma suis* in Slaughter Pigs, with Correlation of PCR Results to Hematological Findings. Vet. Microbiol..

[6] Wang X., Cui Y., Zhang Y., Shi K., Yan Y., Jian F., Zhang L., Wang R., Ning C. (2017). Molecular Characterization of Hemotropic Mycoplasmas (Mycoplasma ovis and ‘Candidatus Mycoplasma haemovis’) in Sheep and Goats in China. BMC Vet. Res..

[7] Messick J.B. (2004). Hemotrophic Mycoplasmas (Hemoplasmas): A Review and New Insights into Pathogenic Potential. Vet. Clin. Pathol..

[8] Stadler J., Ade J., Hermanns W., Ritzmann M., Wentzel S., Hoelzle K., Hoelzle L.E. (2021). Clinical, Haematological and Pathomorphological Findings in Mycoplasma suis-Infected Pigs. BMC Vet. Res..

[9] Maggi R.G., Compton S.M., Trull C.L., Mascarelli P.E., Mozayeni B.R., Breitschwerdt E.B. (2013). Infection with Hemotropic Mycoplasma Species in Patients with or without Extensive Arthropod or Animal Contact. J. Clin. Microbiol..

[10] Hoelzle L.E., Adelt D., Hoelzle K., Heinritzi K., Wittenbrink M.M. (2003). Development of a Diagnostic PCR Assay Based on Novel DNA Sequences for the Detection of Mycoplasma suis (Eperythrozoon suis) in Porcine Blood. Vet. Microbiol..

[11] Santana M.D.S., Hoppe E.G.L., Carraro P.E., Calchi A.C., de Oliveira L.B., do Amaral R.B., Mongruel A.C.B., Machado D.M.R., Burger K.P., Barros-Batestti D.M. (2022). Molecular Detection of Vector-Borne Agents in Wild Boars (Sus scrofa) and Associated Ticks from Brazil, with Evidence of Putative New Genotypes of Ehrlichia, Anaplasma, and Haemoplasmas. Transbound. Emerg. Dis..

[12] Gatto I.R.H., Sonálio K., Amaral R.B., Morés N., Dalla Costa O.A., André M.R., de Oliveira L.G. (2019). High Frequency and Molecular Characterization of Porcine Hemotrophic Mycoplasmas in Brazil. Vet. Microbiol..

[13] Thongmeesee K., Sri-in C., Kaewthamasorn M., Thanee S., Wattanaphansak S., Tiawsirisup S. (2023). Establishment of Molecular Diagnostics Targeting the 23S Ribosomal RNA Gene for the Detection of *Mycoplasma suis* Infection in Thai Domestic Pigs. Acta Trop..

[14] Stadler J., Ade J., Ritzmann M., Hoelzle K., Hoelzle L.E. (2020). Detection of a Novel Haemoplasma Species in Fattening Pigs with Skin Alterations, Fever and Anaemia. Vet. Rec..

[15] Brasil Secretaria de Defesa Agropecuária. Instrução Normativa no 44, de 4 de dezembro de 2017. Estabelece as normas para a Certificação Sanitária da Compartimentação da Cadeia Produtiva de Suínos. Diário Oficial da República Federativa do Brasil, Brasília, 19 December 2017. https://www.gov.br/agricultura/pt-br/assuntos/sanidade-animal-e-vegetal/saude-animal/programas-de-saude-animal/sanidade-suidea/legislacaosuideos/2017IN44de19dedezembrode2017COMPARTIMENTAO_SUNOS.pdf/view.

[16] Varanat M., Maggi R.G., Linder K.E., Breitschwerdt E.B. (2011). Molecular Prevalence of Bartonella, Babesia, and Hemotropic Mycoplasma Sp. In Dogs with Splenic Disease. J. Vet. Intern. Med..

[17] Cruz N.R.N., André M.R., Baraldi T.G., Mathias L.A., Braz L.A.N., Oliveira L.G., Santana A.E. (2023). Molecular Prevalence of Mycoplasma parvum in Production Cycle of Technified Swine Herds. Arq. Bras. Med. Vet. Zootec..

[18] Zhongyang L., Jiansong Z., Yijuan S., Yuting X., Yufeng L., Jiarong X. (2017). Seroprevalence of *Mycoplasma suis* Infection in Pigs in Eastern China as Estimated by a Blocking Enzyme-Linked Immunosorbent Assay. Can. J. Vet. Res..

[19] Ngo T.N.T., Nguyen N.M., Thanawongnuwech R., Thong L.M., Nguyen T.P.T., Nguyen T.T., Do D.T. (2024). Coinfection of *Mycoplasma suis* and Porcine Circovirus Type 3 Is Linked to Reproductive Failure in Pig Farms. Vet. World.

[20] Brissonnier M., Normand V., Lebret A., Moalic P.-Y., Guyomard A.-S., Bachy V., Berton P., Auvigne V., Bouchet F., Boulbria G. (2020). Frequency of Infection with *Mycoplasma suis* in Gestating Sows Using qPCR on Ten Commercial French Herds, and Impact of the Infection on Clinical, Haematological and Biochemical Parameters. Porcine Health Manag..

[21] Ade J., Hoelzle K., Stadler J., Ritzmann M., Hoelzle L.E. (2022). Occurrence of Mycoplasma parvum in German Pigs of Different Age Groups Using a Novel Quantitative Real-Time PCR Assay. Pathogens.

[22] Ade J., Eddicks M., Ritzmann M., Hoelzle K., Hoelzle L.E., Stadler J. (2024). Haemotrophic Mycoplasmas Infecting Pigs: A Review of the Current Knowledge. Microorganisms.

[23] Guimarães A.M.S., Biondo A.W., Lara A.C., Messick J.B. (2007). Exploratory Study of *Mycoplasma suis* (Eperythrozoon suis) on Four Commercial Pig Farms in Southern Brazil. Vet. Rec..

[24] Stadler J., Willi S., Ritzmann M., Eddicks M., Ade J., Hoelzle K., Hoelzle L.E. (2019). Detection of *Mycoplasma suis* in Pre-Suckling Piglets Indicates a Vertical Transmission. BMC Vet. Res..

[25] Do D.T.T., Ngo T.T.N., Nguyen N.M., Bui N.T.T., Do D.T. (2024). Case Report of Reproductive Failure and Dermatitis Occurred by PCV3 and *Mycoplasma suis* in Sows at a Breeding Farm. Vet. Integr. Sci..

